# Association between the cholesterol-high-density lipoprotein-glucose (CHG) index and albuminuria risk in diabetic patients

**DOI:** 10.3389/fendo.2026.1937841

**Published:** 2026-10-01

**Authors:** Yuren Zhang, Jinting Xu, Yiping Zhang, Yinan Chen, Qiaolan Liu

**Affiliations:** Department of Endocrinology & Metabolism, Jinjiang Municipal Hospital, Shanghai Sixth People’s Hospital Fujian, Quanzhou, Fujian, China

**Keywords:** albuminuria, cholesterol-high-density lipoprotein-glucose index, glucolipid metabolic disorders, nonlinear relationship, type 2 diabetes mellitus

## Abstract

**Introduction:**

The Cholesterol-High-Density Lipoprotein-Glucose (CHG) index is a novel composite metabolic biomarker. We aimed to evaluate the relationship between the CHG index and albuminuria prevalence in patients with type 2 diabetes mellitus (T2DM).

**Methods:**

A total of 1842 T2DM patients at Jinjiang Municipal Hospital were enrolled in this cross-sectional study. Albuminuria was defined as a urinary albumin-to-creatinine ratio (UACR) > 30 mg/g. Multivariable logistic regression, restricted cubic spline modeling, subgroup analyses, and receiver operating characteristic (ROC) curve analysis were performed to evaluate the independent association.

**Results:**

Patients with albuminuria (n=324) had higher CHG index values. After full adjustment, an elevated CHG index was significantly associated with higher odds of albuminuria (*OR=2.097, 95%CI:1.438-3.060, P<0.001*). Restricted cubic spline analysis revealed a J-shaped nonlinear relationship, with an inflection point at 6.402. The association was more pronounced in patients with hypertension. The CHG index demonstrated an area under curve (AUC) of 63.28% for distinguishing albuminuria, outperforming other metabolic indices.

**Conclusions:**

The CHG index was nonlinearly associated with albuminuria prevalence in patients with T2DM; however, its cross-sectional design precludes causal inference.

## 1 Introduction

The escalating global burden of diabetes has become a critical public health crisis, posing a significant threat to human well-being (1). In diabetic patients, albuminuria - defined as abnormally elevated urinary albumin excretion - serves as both an early biomarker of diabetic kidney disease (DKD) and a surrogate marker of systemic endothelial dysfunction (2–4). It is independently associated with progressive renal function decline, heightened risk of cardiovascular events, and increased mortality (5, 6).

As an emerging composite biomarker, the Cholesterol, High-density Lipoprotein, and Glucose (CHG) index integrates total cholesterol (TC), high-density lipoprotein cholesterol (HDL), and fasting blood glucose (FBG), enabling a comprehensive evaluation of lipid and glucose metabolism alongside insulin sensitivity (7). By aggregating these three core metabolic dimensions, the CHG index facilitates a more holistic characterization of cardiometabolic risk (8, 9). Accumulating evidence also supports its utility in predicting outcomes across a spectrum of conditions-including diabetes, metabolic dysfunction-associated steatotic liver disease (MASLD), cardiovascular and cerebrovascular diseases, and mortality (7, 10–14). This present study aimed to evaluate the association between the CHG index and the prevalence of albuminuria in a cohort of T2DM adults.

## 2 Materials and methods

### 2.1 Study population

This study analyzed 1842 T2DM adults admitted to the Department of Endocrinology & Metabolism, Jinjiang Municipal Hospital, between January 2024 and January 2026. The cohort included 1089 men and 753 women, all of whom met the American Diabetes Association diagnostic criteria for T2DM. Patients were excluded if they had diabetes subtypes other than T2DM; diabetic ketoacidosis; hyperosmolar hyperglycemic state; severe hypoglycemia; acute diabetic infection; diabetic coma; acute cardiovascular or cerebrovascular events; acute kidney injury; severe chronic infectious diseases; malignancy; severe hepatic or renal dysfunction; or autoimmune disorders. This study was conducted in accordance with the Declaration of Helsinki and was approved by the Ethics Committee of Jinjiang Municipal Hospital (Shanghai Sixth People’s Hospital Fujian) (Approval No. jsyyll-2026-132).

### 2.2 CHG index calculation and albuminuria definitions

The CHG index was computed using the formula Ln [total cholesterol (TC, mg/dL) × fasting blood glucose (FBG, mg/dL)/(2×high-density lipoprotein cholesterol (HDL, mg/dL))] and served as the exposure variable (7). To ensure consistency of units, TC (mg/dL) = TC (mmol/L) × 38.67, HDL (mg/dL) = HDL (mmol/L) × 38.67, and FBG (mg/dL) = FBG (mmol/L) × 18.0. Urinary albumin-to-creatinine ratio (UACR) was determined in spot urine specimens. Urinary albumin concentration was measured by immunoturbidimetry (mg/L), and urinary creatinine concentration was quantified using the enzymatic method (g/L). The UACR (mg/g) was calculated as the ratio of urinary albumin to urinary creatinine. Albuminuria was defined as a UACR exceeding 30 mg/g.

### 2.3 Data collection

Anthropometric data, including height and body weight, were obtained using standardized protocols and equipment by experienced healthcare staff. Body mass index (BMI, kg/m^2^) was calculated as weight in kilograms divided by height in meters squared. Demographic information regarding age, gender, smoking history, and alcohol use was recorded. Smoking history was categorized as ever smoking (current or former smoking) versus never smoking. Alcohol use was categorized as current or previous regular consumption versus no alcohol intake. Venous blood specimens were collected after an 8- to 12-hour overnight fast from all patients with diabetes on the morning following admission. FBG was measured using the glucose oxidase method, and hemoglobin A1c (HbA1c) was quantified using high-performance liquid chromatography. Triglycerides (TG), TC, HDL, low-density lipoprotein cholesterol (LDL), alanine aminotransferase (ALT), aspartate aminotransferase (AST), and serum creatinine (Scr) were determined using a Beckman AU5800 automated biochemical system. The 2021 Chronic Kidney Disease Epidemiology Collaboration (CKD-EPI) formula, integrating age, gender, and Scr levels, was used to estimate glomerular filtration rate (eGFR). Hyperlipidemia was defined based on the criteria established by the Adult Treatment Panel III (ATP III) of the National Cholesterol Education Program (NCEP) (15), or by current use of lipid-lowering medication. Hypertension is defined as systolic blood pressure (SBP) ≥140 mmHg and/or diastolic blood pressure (DBP) ≥90 mmHg, a self-reported history of hypertension, or current use of antihypertensive medication.

### 2.4 Statistical analysis

Descriptive statistics for continuous variables are reported as mean ± standard deviation (SD) or median (interquartile range), depending on normality; categorical variables are presented as counts and percentages. Between-group differences were assessed via the Kruskal-Wallis test for continuous measures and the chi-square test for categorical measures. Logistic regression was used to evaluate associations between the CHG index and albuminuria prevalence. Variance inflation factors (VIFs) were computed to detect multicollinearity. A *post hoc* SHapley Additive exPlanations (SHAP) framework was adopted to characterize the contributions of the CHG index and other covariates to albuminuria prevalence, based on the results of the fitted multivariable logistic regression model (16). Nonlinear associations were explored with restricted cubic splines (RCS), and piecewise regression identified cutoff points where risk slopes changed. Prespecified subgroup analyses were performed with adjustment for confounding factors to assess the consistency. The discriminatory capacity of the CHG×BMI, CHG index, the triglyceride-glucose (TyG) index, calculated as ln [TG (mg/dL) × FBG (mg/dL)/2], and the TG/HDL ratio for albuminuria was evaluated using receiver operating characteristic (ROC) curve analysis. All analyses were conducted in R, and statistical significance was defined as a two-sided P value below 0.05.

## 3 Results

### 3.1 Baseline characteristics of T2DM patients

Of the 1842 participants enrolled, 324 (17.6%) presented with albuminuria, while 1518 (82.4%) did not (**Table 1**). Compared with the non-albuminuria group, participants with albuminuria were significantly older (median age: 59 vs. 55 years, *P<0.001*) and exhibited a higher prevalence of hypertension (*P<0.001*), along with lower alcohol use (*P=0.017*). Gender, smoking status, BMI, hyperlipemia, HbA1c, LDL, and FBG did not differ significantly between groups (all *P>0.05*). Biochemically, the albuminuria group demonstrated lower ALT and AST levels yet higher TG, TC, and Scr, alongside reduced HDL and eGFR (all *P<0.05*). The CHG index was also significantly elevated in the albuminuria group (6.12 ± 0.64 vs. 6.03 ± 0.56, *P=0.011*). When stratified by CHG quartiles (Q1-Q4), participants in higher quartiles were progressively younger and predominantly male (**Table 2**) (both *P<0.001*). Smoking history, alcohol use, BMI, and Scr increased stepwise across quartiles, as did HbA1c, AST, ALT, TG, TC, LDL, and the prevalence of hyperlipemia (all *P<0.01*). Conversely, HDL, eGFR, and hypertension prevalence decreased (both *P<0.001*). The proportion of albuminuria was highest in Q4 group (21.48%) relative to Q1 group (14.75%, *P=0.005*).

**Table 1:** Baseline characteristics of patients with type 2 diabetes mellitus, stratified by albuminuria status.

Variables	Overall (N=1842)	Non-albuminuria (N=1518)	Albuminuria (N=324)	*P* value
Age (years)	56.00 (46.00-66.00)	55.00 (45.00-66.00)	59.00 (50.00-69.00)	<0.001
Gender, n (%)				0.580
Female	753 (40.88%)	625 (41.17%)	128 (39.51%)	
Male	1089 (59.12%)	893 (58.83%)	196 (60.49%)	
Smoking history, n (%)				0.766
No	1467 (79.64%)	1207 (79.51%)	260 (80.25%)	
Yes	375 (20.36%)	311 (20.49%)	64 (19.75%)	
Alcohol use, n (%)				0.017
No	1629 (88.44%)	1330 (87.62%)	299 (92.28%)	
Yes	213 (11.56%)	188 (12.38%)	25 (7.72%)	
BMI (kg/m2)	24.80 ± 3.19	24.85 ± 3.20	24.56 ± 3.17	0.197
Hypertension, n (%)				<0.001
No	1084 (58.85%)	962 (63.37%)	122 (37.65%)	
Yes	758 (41.15%)	556 (36.63%)	202 (62.35%)	
Hyperlipemia, n (%)				0.399
No	909 (49.35%)	756 (49.80%)	153 (47.22%)	
Yes	933 (50.65%)	762 (50.20%)	171 (52.78%)	
HbA1c (%)	11.00 (8.62-12.09)	11.06 (8.70-12.06)	10.55 (8.30-12.31)	0.619
AST (U/L)	30.03 (19.62-38.08)	31.90 (20.80-38.93)	23.25 (16.40-30.64)	<0.001
ALT (U/L)	34.31 (20.00-47.00)	38.10 (22.10-48.58)	22.66 (15.00-31.68)	<0.001
TG (mmol/L)	2.13 (1.45-3.05)	2.06 (1.43-2.97)	2.38 (1.57-3.62)	0.011
TC (mmol/L)	5.36 (4.66-5.95)	5.33 (4.69-5.92)	5.52 (4.48-6.17)	0.005
HDL (mmol/L)	1.16 (1.03-1.33)	1.16 (1.04-1.33)	1.14 (0.99-1.32)	0.029
LDL (mmol/L)	3.16 (2.68-3.64)	3.15 (2.68-3.62)	3.25 (2.58-3.75)	0.057
FBG (mmol/L)	11.40 (8.29-13.66)	11.48 (8.31-13.56)	11.11 (8.14-14.26)	0.645
Scr (μmoI/L)	64.80 (54.60-75.29)	63.47 (53.60-71.88)	88.22 (63.00-126.62)	<0.001
eGFR (ml/min/1.73 m²)	108.20 (89.81-127.00)	111.00 (97.00-128.87)	76.61 (50.00-110.00)	<0.001
CHG index	6.05 ± 0.58	6.03 ± 0.56	6.12 ± 0.64	0.011

**Table 2:** Baseline characteristics of patients with type 2 diabetes mellitus, stratified by CHG index quartiles.

Variables	Q1	Q2	Q3	Q4	*P* value
Age (years)	63.00 (54.00-71.00)	60.00 (51.00-68.00)	53.00 (44.75-62.00)	46.00 (36.00-57.00)	<0.001
Gender, n (%)					<0.001
Female	246 (53.36%)	221 (48.04%)	182 (39.57%)	104 (22.56%)	
Male	215 (46.64%)	239 (51.96%)	278 (60.43%)	357 (77.44%)	
Smoking history, n (%)					<0.001
No	398 (86.33%)	383 (83.26%)	361 (78.48%)	325 (70.50%)	
Yes	63 (13.67%)	77 (16.74%)	99 (21.52%)	136 (29.50%)	
Alcohol use, n (%)					<0.001
No	428 (92.84%)	416 (90.43%)	401 (87.17%)	384 (83.30%)	
Yes	33 (7.16%)	44 (9.57%)	59 (12.83%)	77 (16.70%)	
BMI (kg/m2)	24.05 ± 2.61	24.08 ± 2.93	24.54 ± 3.13	26.52 ± 3.38	<0.001
HbA1c (%)	8.29 (7.40-9.62)	9.91 (8.39-11.69)	11.59 (10.43-12.21)	12.02 (11.50-12.62)	<0.001
AST (U/L)	20.00 (16.00-27.60)	28.00 (20.52-34.14)	33.69 (24.67-37.73)	39.72 (28.84-56.83)	<0.001
ALT (U/L)	19.50 (14.30-30.40)	30.95 (21.62-39.75)	40.20 (26.66-47.14)	50.80 (33.56-73.12)	<0.001
TG (mmol/L)	1.31 (0.91-1.66)	1.70 (1.38-2.40)	2.32 (1.85-2.94)	3.51 (2.84-4.42)	<0.001
TC (mmol/L)	4.34 (3.45-4.93)	5.23 (4.83-5.58)	5.57 (5.13-5.98)	5.99 (5.81-6.35)	<0.001
HDL (mmol/L)	1.28 (1.13-1.52)	1.20 (1.08-1.35)	1.16 (1.04-1.25)	1.04 (0.94-1.15)	<0.001
LDL (mmol/L)	2.40 (1.78-2.87)	3.08 (2.85-3.33)	3.33 (3.06-3.70)	3.67 (3.43-3.88)	<0.001
FBG (mmol/L)	7.21 (5.76-8.22)	9.67 (8.57-11.42)	12.54 (11.36-13.38)	14.53 (13.71-15.54)	<0.001
Scr (μmoI/L)	62.40 (51.70-76.00)	65.67 (53.08-80.27)	63.65 (54.35-72.32)	65.56 (60.06-74.70)	0.002
eGFR (ml/min/1.73 m²)	114.99 (99.59-131.00)	110.76 (97.36-128.12)	102.28 (83.99-121.73)	102.00 (83.22-124.00)	<0.001
CHG index	5.28 ± 0.35	5.93 ± 0.11	6.28 ± 0.10	6.72 ± 0.27	<0.001
Hypertension, n (%)					<0.001
No	213 (46.20%)	228 (49.57%)	307 (66.74%)	336 (72.89%)	
Yes	248 (53.80%)	232 (50.43%)	153 (33.26%)	125 (27.11%)	
Hyperlipemia, n (%)					<0.001
No	373 (80.91%)	282 (61.30%)	182 (39.57%)	72 (15.62%)	
Yes	88 (19.09%)	178 (38.70%)	278 (60.43%)	389 (84.38%)	
Albuminuria, n (%)					0.005
No	393 (85.25%)	368 (80.00%)	395 (85.87%)	362 (78.52%)	
Yes	68 (14.75%)	92 (20.00%)	65 (14.13%)	99 (21.48%)	

### 3.2 Nonlinear association between the CHG index and albuminuria prevalence

**Table 3** details hierarchical logistic regression models relating the CHG index to the prevalence of albuminuria. The crude model yielded an odd ratio (OR) of 1.317 per unit increase in CHG index (*95%CI:1.064-1.630, P = 0.012*) (Model 1). After controlling for age, gender, smoking history, and alcohol use, the OR increased to *1.646 (95%CI:1.301-2.083, P<0.001*) (Model 2). In Model 3, which additionally adjusted for BMI, hypertension, hyperlipidemia, HbA1c, AST, ALT, Scr and eGFR-the association remained robust (*OR=2.097, 95%CI:1.438-3.060, P<0.001*). The standardized estimate in Model 3 was OR 1.531 per SD (*95%CI:1.232-1.903, P<0.001*). When the CHG index was analyzed by quartiles (Q1-Q4), participants in Q2 group had higher odds (*OR=1.553, 95%CI:1.007-2.395, P = 0.046*), Q3 group did not differ significantly from Q1 group (*OR=1.346, 95%CI:0.798-2.269, P = 0.265*), and Q4 group exhibited markedly elevated odds (*OR=3.084, 95%CI:1.685-5.646, P<0.001*). A J-shaped pattern between the CHG index and albuminuria prevalence was observed through RCS analysis (**Figure 1**; *P-overall<0.001, P-non-linear=0.003*). Piecewise regression subsequently identified a threshold value of 6.402 (**Table 4**). Below the inflection point, the CHG index was not significantly associated with albuminuria prevalence (*OR=1.302, 95%CI:0.842-2.014, P = 0.235*). Above this threshold, each unit increase in CHG corresponded to 8.173-fold higher odds of albuminuria (*OR=8.173, 95%CI: 3.658-18.261, P<0.001*). Additionally, **Figure 2** displays the SHAP-based variable importance ranking, in which the CHG index ranks fourth. Elevated CHG values (in red) cluster in the positive SHAP region, consistent with a positive association with the prevalence of albuminuria.

**Table 3:** Multivariable logistic regression analysis of the association between CHG index and albuminuria.

Variables	OR (95%CI), *P* value		
	Model 1	Model 2	Model 3
Continuous			
CHG index	1.317 (1.064, 1.630) 0.012	1.646 (1.301, 2.083) <0.001	2.097 (1.438, 3.060) <0.001
Each SD increase	1.172 (1.036, 1.325) 0.012	1.332 (1.164, 1.526) <0.001	1.531 (1.232, 1.903) <0.001
Categories			
Quartile 1	1.000 (reference)	1.00 (reference)	1.00 (reference)
Quartile 2	1.445 (1.024, 2.038) 0.036	1.562 (1.103, 2.213) 0.012	1.553 (1.007, 2.395) 0.046
Quartile 3	0.951 (0.659, 1.373) 0.789	1.146 (0.786, 1.672) 0.479	1.346 (0.798, 2.269) 0.265
Quartile 4	1.581 (1.125, 2.221) 0.008	2.209 (1.523, 3.205) <0.001	3.084 (1.685, 5.646) <0.001
P for trend	0.071	<0.001	0.001

OR: odds ratio.

95% CI: 95% confidence interval.

Model 1: Non-adjusted.

Model 2: adjusted for age, gender, smoking history, and alcohol use.

Model 3: adjusted for age, gender, smoking history, alcohol use, BMI, hypertension, hyperlipemia, HbA1c, AST, ALT, Scr, and eGFR.

**Figure 1:**
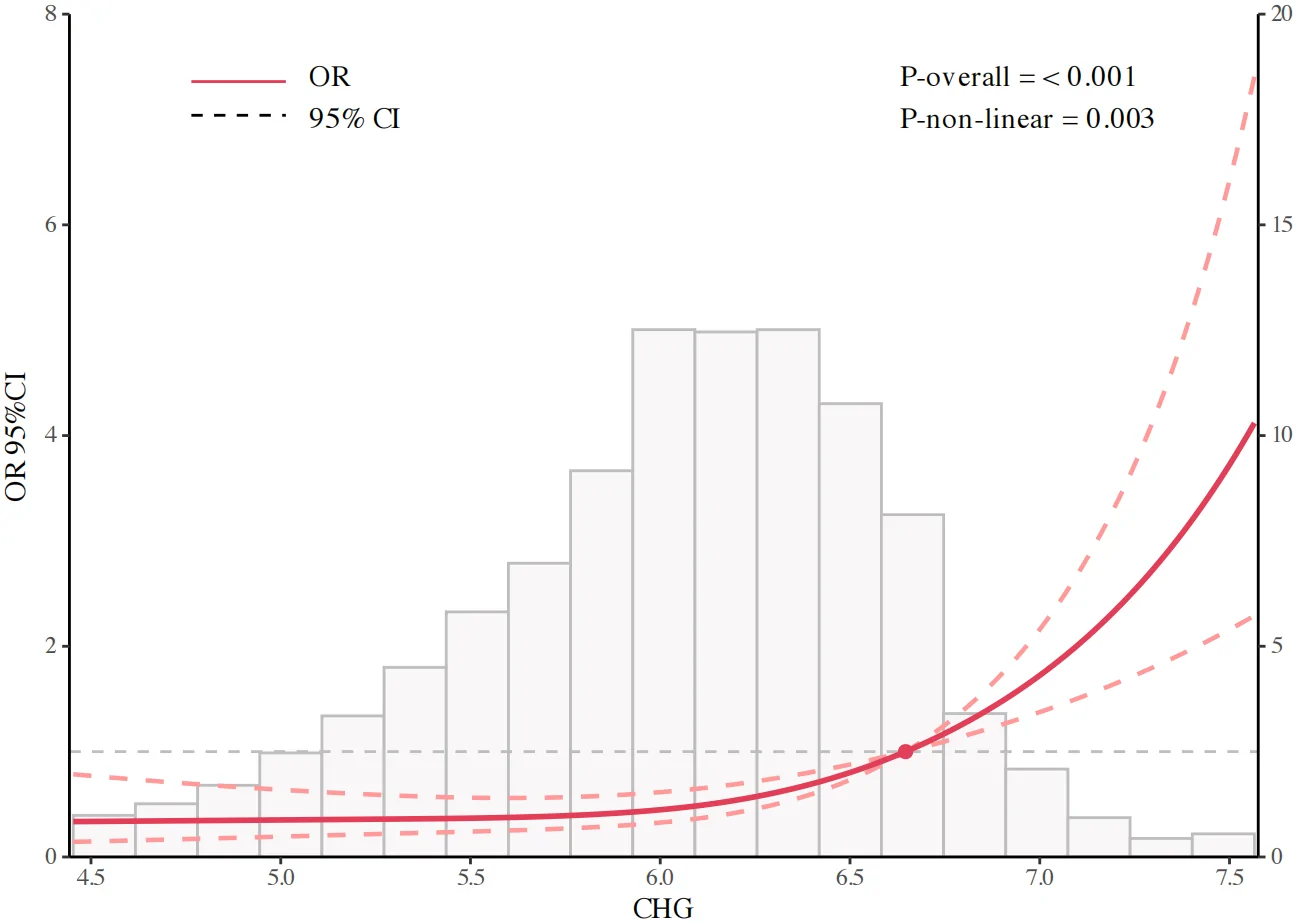
Dose-response relationship between the CHG index and albuminuria.

**Table 4:** Piecewise regression analysis of CHG index and albuminuria.

Model	OR (95% CI), *P* value
Fitting by standard logistic model	
	2.097 (1.438, 3.060) <0.001
Fitting by two-piecewise logistic model	
Breakpoint	6.402
OR1 (<6.402)	1.302 (0.842, 2.014) 0.235
OR2 (>6.402)	8.173 (3.658, 18.261) <0.001
OR2/OR1	6.275 (2.382, 16.530) <0.001
P for logarithmic likelihood ratio	<0.001

adjusted for age, gender, smoking history, alcohol use, BMI, hypertension, hyperlipemia, HbA1c, AST, ALT, Scr, and eGFR.

**Figure 2:**
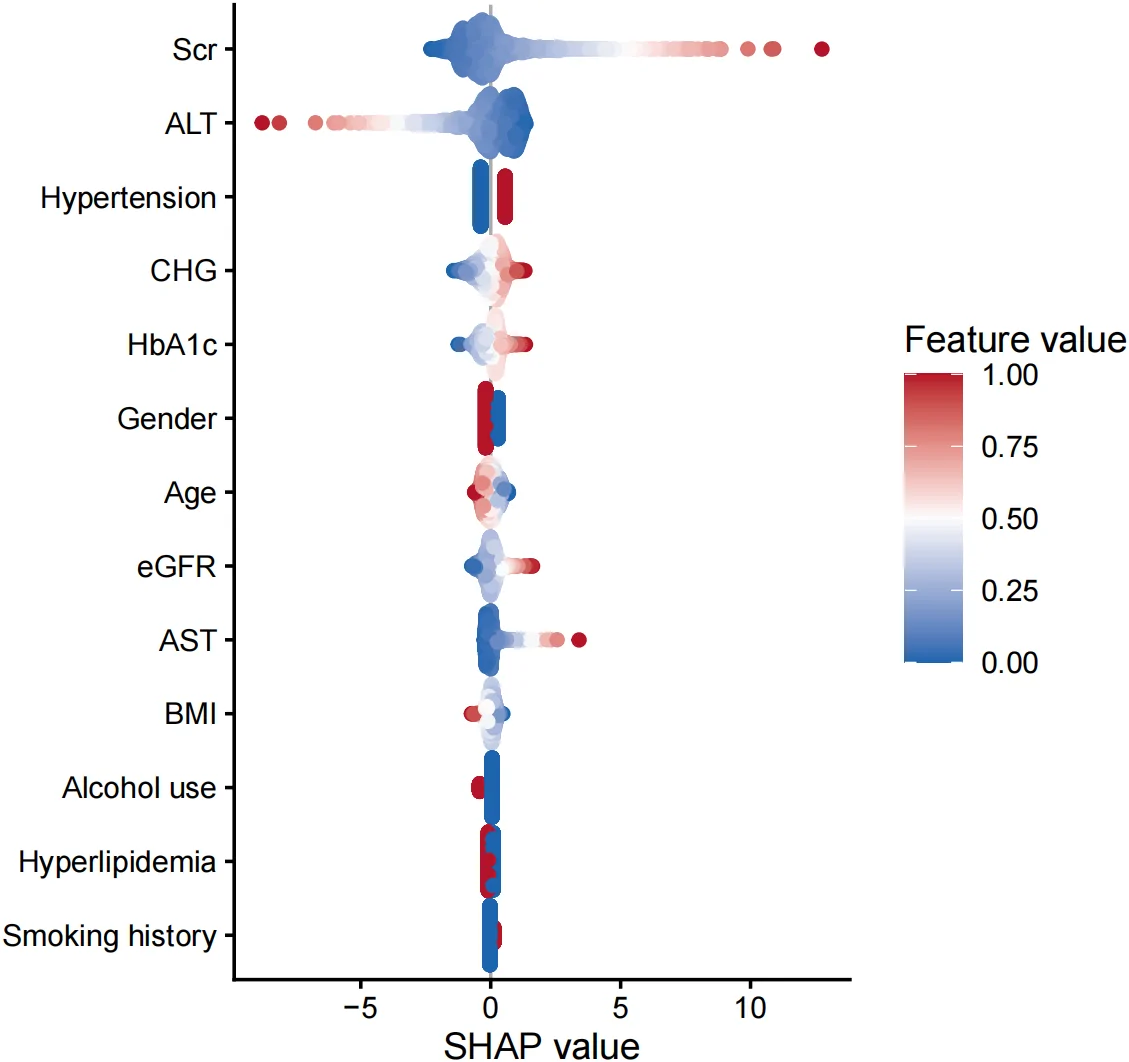
Shapley additive explanations-based feature importance ranking for albuminuria.

### 3.3 Stratified analysis and ROC performance

Subgroup forest plots demonstrated consistent positive associations across all subgroups, including age (<60/≥60 years), gender (female/male), BMI (<24/24-28/≥28 kg/m^2^), eGFR (<60/≥60 ml/min/1.73m²), hypertension (Yes/No), hyperlipidemia (Yes/No), smoking history (Yes/No), and alcohol use (Yes/No) (**Figure 3**). A significant interaction with hypertension was identified (*P for interaction<0.05*), with a stronger relationship among hypertensive (*OR=3.396, 95%CI:1.980-5.824*) compared to non-hypertensive individuals (*OR=1.376, 95%CI:0.822-2.304*), indicating that hypertension status modifies the CHG-albuminuria association. **Figure 4** depicts the ROC curves for the CHG index, the TyG index, and the TG/HDL ratio in assessing their associations with albuminuria in T2DM patients. The CHG×BMI showed the highest area under the curve (AUC) of 69.94%, followed by CHG index (AUC: 63.28%), the TyG index (AUC: 60.71%) and the TG/HDL ratio (AUC: 54.43%).

**Figure 3:**
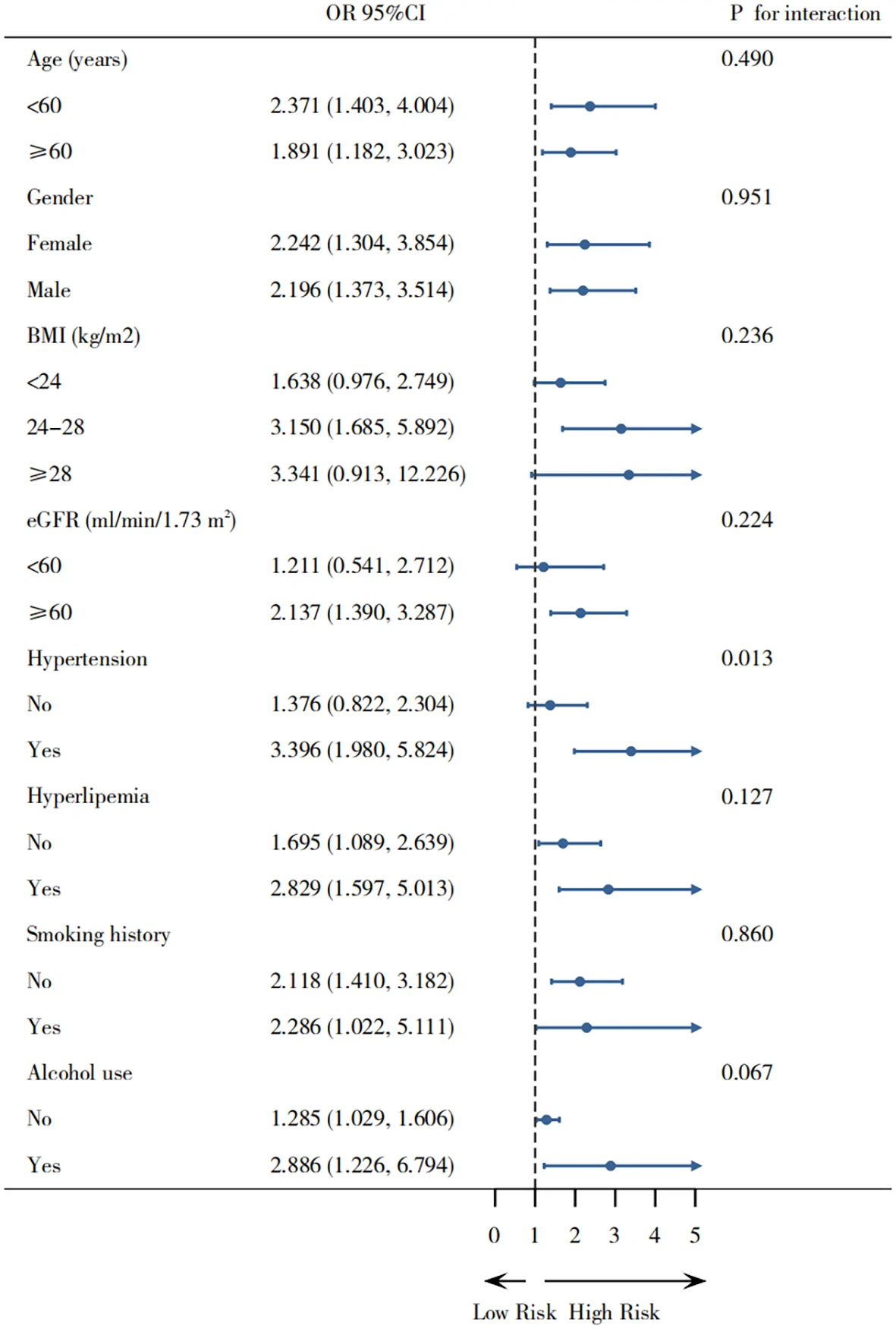
Subgroup analysis of the CHG index-albuminuria association.

**Figure 4:**
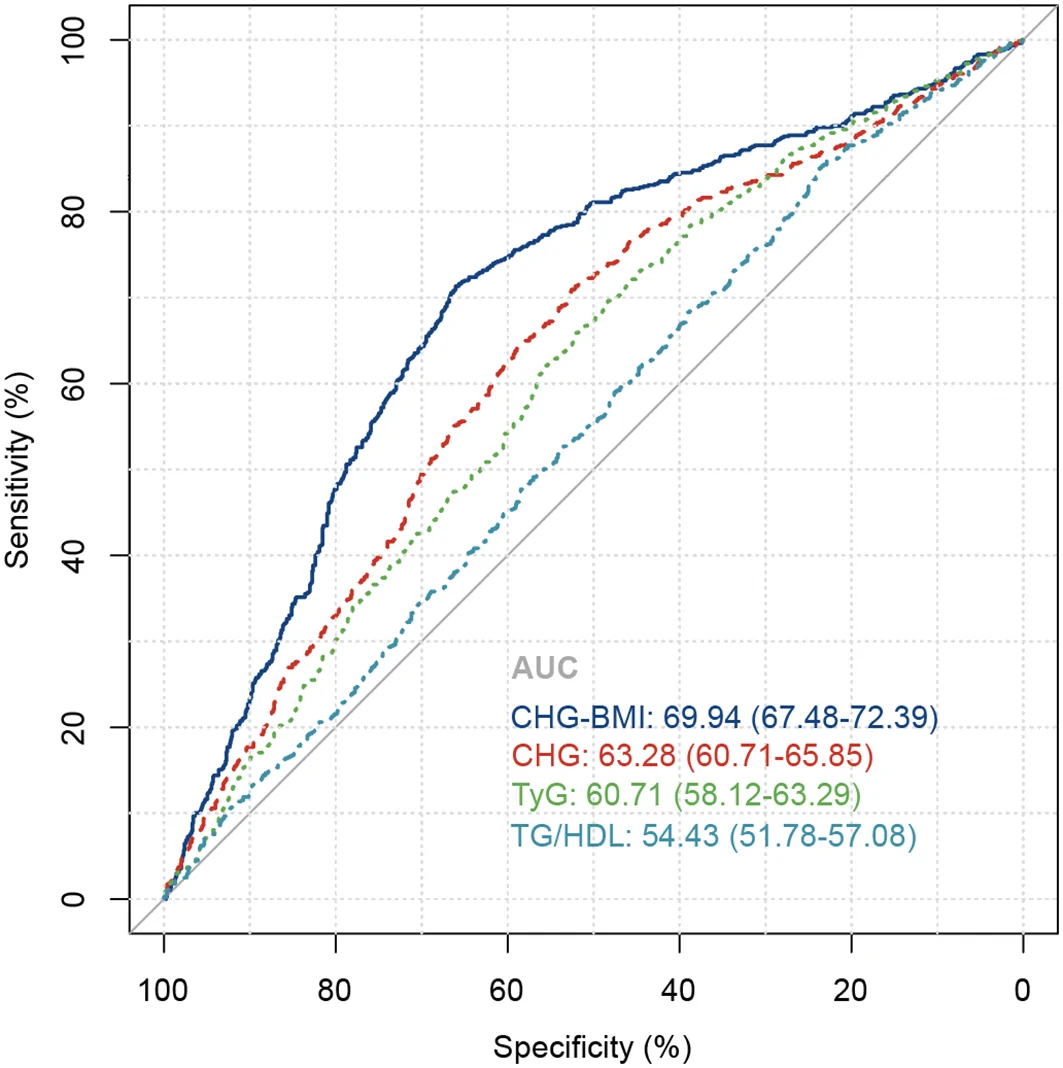
Receiver operating characteristic curves for discriminating albuminuria.

## 4 Discussion

This study examined the relationship between the CHG index and the prevalence of albuminuria in T2DM patients. After full adjustment for confounders, a higher CHG index was independently and positively associated with the prevalence of albuminuria. This association followed a nonlinear J-shaped pattern, with a threshold of 6.402. The CHG index may serve as a simple and readily accessible metabolic indicator.

As a composite biomarker that simultaneously captures glucose and lipid metabolic status, the CHG index has been shown in previous studies to outperform conventional metrics in cardiometabolic risk assessment (9, 17, 18). Building on this evidence, the present study extends the application of the CHG index to the domain of DKD. The CHG index demonstrated superior discriminatory performance in albuminuria relative to the TyG index and the TG/HDL ratio. Although the TyG index is a widely used surrogate marker of insulin resistance based primarily on fasting TG and glucose levels, it does not capture cholesterol-related abnormalities or HDL deficiency. Similarly, the TG/HDL ratio reflects the balance between triglyceride-rich lipoproteins and HDL but does not account for glycemic burden. Mechanistically, sustained hyperglycemia and elevated free fatty acids activate NF-κB and NLRP3 inflammasome signaling within the kidney, driving the release of pro-inflammatory mediators, and resulting in microvascular endothelial dysfunction, podocyte impairment, and interstitial fibrosis (19, 20). Insulin resistance has been consistently associated with elevated urinary albumin excretion (21). Podocyte injury constitutes the earliest event in the pathogenesis of albuminuria, and insulin signaling in podocytes plays a critical role in maintaining the integrity of the glomerular filtration barrier (22). The combination of hyperglycemia and dyslipidemia also potentiates oxidative stress through augmented reactive oxygen species generation and attenuated antioxidant defense, ultimately leading to glomerular basement membrane thickening, mesangial matrix accumulation, and tubular epithelial cell apoptosis (23, 24). The J-shaped association suggests that the pathophysiological impact of combined glucose-lipid dysregulation on renal function may follow a biphasic course, wherein modest metabolic derangements are adequately buffered by endogenous compensatory mechanisms, whereas once a critical metabolic load is exceeded, renal vulnerability accelerates disproportionately. Additionally, stratified analyses showed a stronger association in participants with hypertension, possibly reflecting the combined effects of metabolic dysregulation and hypertensive hemodynamic stress on renal microvascular injury.

Several limitations should be acknowledged. First, the cross-sectional design precludes causal inference. Second, albuminuria was defined by a single spot UACR measurement, although Kidney Disease: Improving Global Outcomes (KDIGO) guidelines recommend confirmation with at least two abnormal measurements over 3–6 months. Third, information on diabetes duration and the use of antidiabetic and reno protective medications was incomplete. Finally, the population-specific inflection point represents a statistical turning point rather than a validated diagnostic or clinical threshold and requires confirmation in prospective, multicenter studies.

## 5 Conclusion

The CHG index is a practical metabolic marker associated with albuminuria prevalence in patients with T2DM.

## Data Availability

The raw data supporting the conclusions of this article will be made available by the authors, without undue reservation.

## References

[1] Sun H Saeedi P Karuranga S Pinkepank M Ogurtsova K Duncan BB . IDF Diabetes Atlas: global, regional and country-level diabetes prevalence estimates for 2021 and projections for 2045. Diabetes Res Clin Pract. (2022) 183:109119. doi: 10.1016/j.diabres.2021.109119 34879977 PMC11057359

[2] Raja P Maxwell AP Brazil DP . The potential of albuminuria as a biomarker of diabetic complications. Cardiovasc Drugs Ther. (2021) 35:455–66. doi: 10.1007/s10557-020-07035-4 32681438 PMC8105227

[3] Martens RJH Houben A Kooman JP Berendschot T Dagnelie PC van der Kallen CJH . Microvascular endothelial dysfunction is associated with albuminuria: the Maastricht Study. J Hypertens. (2018) 36:1178–87. doi: 10.1097/hjh.0000000000001674 29373478

[4] Jung CY Yoo TH . Pathophysiologic mechanisms and potential biomarkers in diabetic kidney disease. Diabetes Metab J. (2022) 46:181–97. doi: 10.4093/dmj.2021.0329 35385633 PMC8987689

[5] Claudel SE Verma A . Albuminuria in cardiovascular, kidney, and metabolic disorders: a state-of-the-art review. Circulation. (2025) 151:716–32. doi: 10.1161/circulationaha.124.071079 40063723 PMC11902889

[6] Barzilay JI Farag YMK Durthaler J . Albuminuria: an underappreciated risk factor for cardiovascular disease. J Am Heart Assoc. (2024) 13:e030131. doi: 10.1161/jaha.123.030131 38214258 PMC10926810

[7] Mansoori A Nosrati M Dorchin M Mohammadyari F Derakhshan-Nezhad E Ferns G . A novel index for diagnosis of type 2 diabetes mellitus: cholesterol, high density lipoprotein, and glucose (CHG) index. J Diabetes Investig. (2025) 16:309–14. doi: 10.1111/jdi.14343 39569998 PMC11786182

[8] Mo D Zhang P Zhang M Dai H Guan J . Cholesterol, high-density lipoprotein, and glucose index versus triglyceride-glucose index in predicting cardiovascular disease risk: a cohort study. Cardiovasc Diabetol. (2025) 24:116. doi: 10.1186/s12933-025-02675-y 40065297 PMC11895360

[9] Zhang Y Liu B Zhu Y Xie Y Du Y Xiong P . Associations of cumulative exposure and dynamic trajectories of cholesterol-HDL-glucose (CHG) index with cardiovascular disease in middle-aged and older Chinese adults: a longitudinal analysis. Cardiovasc Diabetol. (2026) 25. doi: 10.1186/s12933-026-03137-9 41832529 PMC12994255

[10] Pan B Lv X Guo X Liu J . Nonlinear relationship between the CHG index and MASLD in type 2 diabetes mellitus: mediating role of immunoinflammatory markers. Postgrad Med. (2026) 138:345–56. doi: 10.1080/00325481.2026.2661571 42021546

[11] Chen J Liang Q Chen Z Liu W Chen X Li H . Evaluation of the CHG index for identifying metabolic dysfunction associated steatotic liver disease: evidence from two independent Asian non-obese populations. Front Nutr. (2026) 13:1757164. doi: 10.3389/fnut.2026.1757164 41798832 PMC12963058

[12] Zhang Z Chen M Cai D Fan M Wang Y . Association between the cholesterol, high-density lipoprotein, and glucose index and type 2 diabetes mellitus prevalence in US adults: a cross-sectional study based on National Health and Nutrition Examination Survey 2009-2018. J Int Med Res. (2025) 53:3000605251375557. doi: 10.1177/03000605251375557 40937624 PMC12432308

[13] Wei S Yu S Qian C Xu Z Jia Y Chen B . Exploring the U-shaped nonlinear relationship of CHG index with metabolic syndrome and mortality risks in metabolic syndrome patients. Lipids Health Dis. (2025) 24:294. doi: 10.1186/s12944-025-02714-3 41023729 PMC12482814

[14] Xiao Q Huang W Zhuang Y Chen Y Peng G Li Y . Evaluating the role of CHG index in predicting stroke risk among adults with varying glucose regulation. Aging Clin Exp Res. (2025) 37:334. doi: 10.1007/s40520-025-03243-w 41296126 PMC12657532

[15] Third Report of the National Cholesterol Education Program (NCEP) Expert Panel on Detection, Evaluation, and Treatment of High Blood Cholesterol in Adults (Adult Treatment Panel III) final report. Circulation. (2002) 106:3143–421. doi: 10.1161/circ.106.25.3143 12485966

[16] Tan X Zhang X Chai J Ji W Ru J Yang C . Constructing a predictive model for early-onset sepsis in neonatal intensive care unit newborns based on SHapley Additive exPlanations explainable machine learning. Transl Pediatr. (2024) 13:1933–46. doi: 10.21037/tp-24-278 39649648 PMC11621883

[17] Song Y Chen X Chang Z Bian X He J Li B . The cholesterol, high-density lipoprotein, and glucose (CHG) index as a novel metabolic marker for predicting adverse outcomes in myocardial infarction survivors: insights from two large prospective cohorts. Cardiovasc Diabetol. (2026) 25. doi: 10.1186/s12933-026-03104-4 41731494 PMC12980928

[18] Wang J Tu G Tao Q Li R Zhai H Hong S . The prognostic significance of cholesterol, high-density lipoprotein and glucose (CHG) index in evaluating all-cause mortality risk in metabolic dysfunction-associated steatotic liver disease (MASLD) individuals: evidence from two cohort studies. Cardiovasc Diabetol. (2026) 25. doi: 10.1186/s12933-026-03171-7 41943105 PMC13196152

[19] Wang Y Yang J Chang X Xue Y Liu G Zhang T . Isoliquiritigenin alleviates diabetic kidney disease via oxidative stress and the TLR4/NF-κB/NLRP3 inflammasome pathway. Mol Nutr Food Res. (2024) 68:e2400215. doi: 10.1002/mnfr.202400215 39082076

[20] Tang SCW Yiu WH . Innate immunity in diabetic kidney disease. Nat Rev Nephrol. (2020) 16:206–22. doi: 10.1038/s41581-019-0234-4 31942046

[21] Pilz S Rutters F Nijpels G Stehouwer CD Højlund K Nolan JJ . Insulin sensitivity and albuminuria: the RISC study. Diabetes Care. (2014) 37:1597–603. doi: 10.2337/dc13-2573 24623021

[22] Jauregui A Mintz DH Mundel P Fornoni A . Role of altered insulin signaling pathways in the pathogenesis of podocyte malfunction and microalbuminuria. Curr Opin Nephrol Hypertens. (2009) 18:539–45. doi: 10.1097/MNH.0b013e32832f7002 19724224 PMC2907246

[23] Ma X Ma J Leng T Yuan Z Hu T Liu Q . Advances in oxidative stress in pathogenesis of diabetic kidney disease and efficacy of TCM intervention. Ren Fail. (2023) 45:2146512. doi: 10.1080/0886022x.2022.2146512 36762989 PMC9930779

[24] Su S Ma Z Wu H Xu Z Yi H . Oxidative stress as a culprit in diabetic kidney disease. Life Sci. (2023) 322:121661. doi: 10.1016/j.lfs.2023.121661 37028547

